# Spatial differentiation characteristics of the Hemiptera insects in China

**DOI:** 10.1002/ece3.70180

**Published:** 2024-08-13

**Authors:** Zhipeng Li, Xinyi Zhang, Jian Zhao, Hong Chen, Maofen Tian

**Affiliations:** ^1^ Institute of Digital Agriculture, Fujian Academy of Agricultural Sciences Fuzhou China; ^2^ State Key Laboratory for Ecological Pest Control of Fujian and Taiwan Crops, Institute of Applied Ecology Fujian Agriculture and Forestry University Fuzhou China

**Keywords:** geographical detector, Hemiptera insects, hot spot analysis, influencing factors, spatial autocorrelation, spatial distribution

## Abstract

The Hemiptera insects are the largest incomplete metamorphosis insect group in Insecta and play a vital role in ecosystems and biodiversity. Previous studies on the spatial distribution of Hemiptera insects mainly focused on a specific region and insect, this study explored the spatial distribution characteristics of Hemiptera insects in China (national scale), and further clarified the dominant factors affecting their spatial distribution. We used spatial autocorrelation analysis, hot spot analysis, and standard ellipse to investigate the spatial distribution characteristics of Hemiptera insects in China. Furthermore, we used geographic detectors to identify the main factors affecting their spatial distribution under China's six agricultural natural divisions and explore the influencing mechanism of dominant factors. The results show that: (i) The spatial differentiation characteristics of Hemiptera insects in China are significant, and their distribution has obvious spatial agglomeration. The Hu Huanyong Line is an important dividing line for the spatial distribution of Hemiptera insects in China. From the city scale, the HH type (high‐high cluster) is mainly distributed on both sides of the Hu Huanyong Line. (ii) The hot spots of Hemiptera insects are mainly distributed in southwest China, along the Qinling Mountains, the western side of the Wuyi Mountains, the Yinshan Mountains, the Liupanshan Mountains, the Xuefeng Mountains, the Nanling Mountains, and other mountainous areas. (iii) Under agricultural natural divisions, the influence of natural environmental factors on the spatial distribution of Hemiptera insects is obviously different. Temperature and precipitation are the dominant factors. Natural factors and socio‐economic factors have formed a positive reinforcement interaction mode on the spatial distribution of Hemiptera insects. These can provide the decision‐making basis for biodiversity conservation and efficient pest control.

## Graphical abstract

The spatial differentiation characteristics of Hemiptera insects in China are significant, and their distribution has obvious spatial agglomeration. Natural factors and socio‐economic factors have formed a positive reinforcement interaction mode on the Hemiptera insects' distribution.

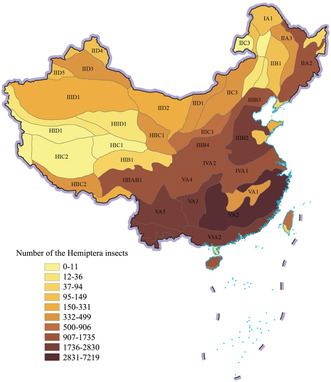

## 1 INTRODUCTION

Insects are important biological resources, which play an important role in protecting biodiversity and maintaining ecological balance and human development (Misof et al., 2014; Ouyang et al., 2019; Yang & Gratton, 2014). In recent years, the biodiversity of insects has been severely threatened by habitat loss, degradation and fragmentation, excessive use of fertilizers and pesticides, biological invasion, climate change, and other factors (Stork, 2018; Wagner, 2019). Therefore, understanding the spatial distribution pattern of insects is of great significance for biodiversity conservation. Hemiptera is the largest incomplete insect group in Insecta, with more than 100,000 known species and more than 11,000 species recorded in China (Li et al., 2017; Shen et al., 2015; Stork, 2018). Many Hemiptera insects are phytophagous insects with a wide range of hosts and are important agricultural and forestry pests (Forero, 2008). Its persistent transmission of plant viruses can lead to the outbreak of plant virus diseases (Wang et al., 2022). In recent years, the research on the macroscale of Hemiptera insects has attracted much attention (Li, Li, et al., 2021; Li, Liu, et al., 2021; Li, Zheng, & Wang, 2021). However, its spatial distribution characteristics such as spatial agglomeration and distribution trend at the national scale need further study.

Species biodiversity and its influencing factors at the provincial and larger macro spatial scales is one of the key and difficult points in biogeography and ecology (Liu & Tang, 2017). The geographical distribution of the species plays a key role in its formation and development (Pennisi, 2005). Studying the species' spatial distribution in large‐scale patterns is helpful in understanding the status of biological diversity and is of great significance for species protection and rational use (Zhang et al., 2022). Insects are the most prosperous animals (Tihelka et al., 2021), and the research on the insect distribution pattern on large geographical scale has attracted more and more attention (Li & Huang, 2022). Spatial statistical analysis is widely used in the field of entomology, which provides a new way to study the spatiotemporal dynamics of insect populations. For example, the descriptive study on geographical spatial distribution of insects (Liu et al., 2009), the distribution of insect faunal components (Ma et al., 2009), insect habitat risk assessment and potential distribution prediction (Zhao et al., 2019), and geographical study on insect biological characters (Peng et al., 2017). However, the existing research on the distribution of Hemiptera insects mainly focuses on special areas and specific species, and it is relatively a weak field to analyze the spatial distribution characteristics of Hemiptera multigroup insects such as distribution agglomeration area, hot spot area, and distribution pattern on a large scale (Wei et al., 2016; Zhao et al., 2020). These are of great significance for understanding insect biodiversity status and protection.

In this study, we focus on the spatial distribution characteristics of Hemiptera insects at provincial and municipal scales in China. (i) We used spatial autocorrelation tools to analyze the global and local spatial distribution patterns of Hemiptera insects, to clarify their spatial agglomeration and the spatial distribution of hot and cold spots in China. (ii) We used the standard deviation ellipse method to analyze its spatial distribution trend. (iii) We used geographical detectors to analyze the driving factors affecting the spatial distribution of Hemiptera insects. From the perspective of geographical science, the research comprehensively applied spatial statistics and geographic detectors to investigate the spatial distribution characteristics of Hemiptera insects, analyzing the interactive effects of environmental factors on the spatial distribution pattern of Hemiptera insects. The study elucidated the complexity and diversity of factors influencing the spatial distribution of Hemiptera insects. Simultaneously, considering the influence of spatial scale effects, this study analyzed the distribution of Hemiptera insects in different agricultural zones, proposing differentiated management strategies for various regions. The outcome of this study can be conducive to biodiversity conservation and efficient pest control.

## 2 MATERIAL AND METHODS

### 2.1 Data sources

Hemiptera insect data: The data were derived from two aspects. One is the diversity and geographical distribution dataset of Chinese Hemiptera insect published by *Biodiversity Science* in 2021 (Li, Li, et al., 2021; Li, Liu, et al., 2021; Li, Zheng, & Wang, 2021). Another is the survey data of major invasive species dynamic distribution and resource library construction projects from China's National Key Research and Development Program.

Map data: The analysis map data (1: 1 million vector data) was derived from the National Geomatics Center of China (http://www.ngcc.cn/ngcc/). The output map data used the standard map of the Ministry of Natural Resources of China (http://bzdt.ch.mnr.gov.cn/). The map approval number of the provincial scale was GS(2019)1697, with a ratio of 1:60,000,000, and the map approval number of the municipal scale was GS(2016)1585, with a ratio of 1:60,000,000.

Eco‐geographical regionalization data: China's climate is complex and diverse. The famous geographer Zheng Du divided China's ecological geography into 48 sub‐regions, and related resources are published in *Geographic Atlas of China* (Wang & Zuo, 2010). The differences in each sub‐region are reflected in temperature, dry humidity, natural vegetation, and so on. Studying Hemiptera insects from the perspective of eco‐geographical divisions can better reveal the characteristics of regional differences. The data were from the Resource and Environmental Science and Data Center of the Chinese Academy of Sciences (https://www.resdc.cn/Default.aspx).

Climate conditions (such as temperature, precipitation, and accumulated temperature) are important in influencing ecological types, and exerting a significant impact on the spatial patterns of species richness (Quintero & Jetz, 2018). Differences in insect biomass and richness highly depend on the environment (Uhler et al., 2021), and insects are poikilothermic animals and their growth and development are closely related to the environment. Temperature plays a pivotal role in the physiology, reproductive capacity, and endosymbiotic bacteria of insects (Ma & Ma, 2016; Xin et al., 2019). Additionally, agricultural intensification is considered a major driver of insect reduction, with studies indicating that it is rapidly decreasing insect biodiversity (Klink et al., 2020; Newbold et al., 2015). Furthermore, insects possess dispersal and migration abilities and are influenced by natural environments, hosts, and human activities (Chen & Ma, 2010). Based on the biological characteristics and habitat requirements of Hemiptera insects, we chose climate conditions, land use, and socio‐economic factors as the three major categories of environmental factors. Therefore, we selected two major categories of environmental factors: natural environmental factors (including climate conditions and land use) and socio‐economic factors.

The natural environmental factors: annual mean temperature (X1), mean diurnal range (X2), isothermality (X3), temperature seasonality (X4), max temperature of the warmest month (X5), precipitation seasonality (X6), annual precipitation (X7), land use (X8), and elevation (X9). The socio‐economic environmental factors: China's population spatial distribution kilometer grid dataset (X10) and China's GDP (Gross Domestic Product) spatial distribution kilometer grid dataset (X11). X1, X5, X8, X9, X10, and X11 were derived from the Resource and Environmental Science and Data Center of the Chinese Academy of Sciences (https://www.resdc.cn/Default.aspx), and the resolution of elevation (X9) at 90 m. X2, X3, X4, X6, and X7 were derived from the WorldClim data website (Fick & Hijmans, 2017) (http://www.worldclim.org).

### 2.2 Methods

#### 2.2.1 Spatial autocorrelation analysis

Spatial autocorrelation is a statistical method used to measure the distribution characteristics and interrelationships of spatial data. There may be some dependency or similarity between data values at locations that are adjacent or close to each other in space, and this dependency or similarity will weaken or disappear with increasing distance. Spatial autocorrelation can be divided into two types: global spatial autocorrelation and local spatial autocorrelation, which are used to describe the spatial distribution pattern of the whole research area and the spatial heterogeneity of the local area, respectively (Cao, 2015). Spatial autocorrelation coefficient is the basic measure of spatial autocorrelation analysis, which is used to measure the aggregation in spatial units (Getis & Aldstadt, 2004). According to the scope of research objects, it can be divided into global spatial autocorrelation and local spatial autocorrelation (Li, Li, et al., 2021; Li, Liu, et al., 2021; Li, Zheng, & Wang, 2021). In this study, the global spatial autocorrelation analysis is used to clarify the spatial distribution of Hemipteran insects in the whole study area, and the local spatial autocorrelation analysis is used to judge the aggregation of Hemipteran insects in a small area. The combination of the global and local spatial autocorrelation analysis can better reflect the spatial distribution and spatial correlation of Hemipteran insects from the large spatial scale and the small–medium spatial scale.

- The global spatial autocorrelation

The Global Moran's *I* index, the Global Getis's C coefficient, and the Global Getis's G coefficient are the commonly used global spatial autocorrelation indicators (Li et al., 2016). The Global Moran's *I* index is the most widely used and can well reflect the distribution pattern between spatial objects (Li, Li, et al., 2021, Li, Liu, et al., 2021, Li, Zheng, & Wang, 2021). The calculation formula is as follows (Cliff & Ord, 1981):

(1) $$I=\frac{n\sum_{i=1}^{n}\sum_{j=1}^{n}w_{\mathrm{ij}}\left(x_{i}-\bar{x}\right)\left(x_{j}-\bar{x}\right)}{\left(\sum_{i=1}^{n}\sum_{j=1}^{n}w_{\mathrm{ij}}\right)\sum_{i=1}^{n}{\left(x_{i}-\bar{x}\right)}^{2}}$$

In the formula, “*n*” represents the number of spatial units (such as province or city). “*w*_*ij*_” is a weight matrix element to measure the spatial relationship of Hemiptera insects, which is used to reflect the correlation of the spatial position of Hemiptera insects. If the study areas are adjacent, *w*_*ij*_ = 1, otherwise *w*_*ij*_ = 0. “*x*_*i*_” is the number of Hemiptera insects in the region numbered “*i*”. “*x*” represents the average number of Hemiptera insects. The value range of Moran's *I* coefficient is [−1,1]. If Moran's *I* coefficient is negative, it means that there is a negative spatial correlation; the Hemiptera insects in adjacent areas have no spatial agglomeration. If the Moran's *I* coefficient is equal to 0, it means that there is no spatial correlation; the Hemiptera insects are randomly distributed. If the Moran's *I* coefficient is positive, it indicates that there is spatial aggregation, and the larger the Moran's *I* value, the more significant the spatial aggregation.

For the Global Moran's *I*, the *Z*‐score was used to test the significance of the spatial autocorrelation of Hemiptera insects. The formula is as follows:

(2) $$Z=\frac{I-E\left(I\right)}{\sqrt{{\mathrm{Var}}_{n}\left(I\right)}}$$

When the *p*‐value is at the 5% significance level: if the *Z*‐score greater than 1.96, it indicates that the spatial distribution of Hemiptera insects in the study area has a significant correlation and tend to spatial agglomeration; if the *Z*‐score within [−1.96, 1.96], it indicates that the spatial distribution correlation of Hemiptera insects is weak; if the *Z*‐score less than −1.96, it indicates that the Hemiptera insect spatial distribution tends to be dispersed.

ii The local spatial autocorrelation

In this study, we used the Local Moran's *I* coefficient to analyze the local spatial autocorrelation of Hemiptera insects. The formula is (Anselin, 1995):

(3) $$I_{I}=\frac{y_{j}-\bar{y}}{S^{2}}\sum_{j=1}^{n}w_{\mathrm{ij}}\left(y_{j}-\bar{y}\right)$$

(4) $$S^{2}=\sum_{j=1,j\neq i}^{n}{\left(y_{j}-\bar{y}\right)}^{2}$$

In the formula, “*n*” represents the number of spatial units (such as province or city). “*w*_*ij*_” is a weight matrix element to measure the Hemiptera insect spatial relationship. “*y*_*j*_” is the Hemiptera insect number in the region numbered *j*. “*ȳ*” represents the average number of Hemiptera insects. The test method for the Local Moran's *I* coefficient is the same as Equation (2).

#### 2.2.2 Hot spot analysis

Hot spot analysis is a method of the local spatial autocorrelation analysis to identify the local region clustering characteristics (hot or cold spots) (Nikitopoulos et al., 2018). In this study, we used the Getis‐Ord Gi index and the calculation formula (Cliff & Ord, 1981) is as follows:

(5) $$Z\;\left(G_{i}\right)=\frac{\sum_{j=1}^{n}w_{\mathrm{ij}}x_{j}-\bar{X}\sum_{j=1}^{n}w_{\mathrm{ij}}}{S\sqrt{\frac{n\sum_{j=1}^{n}{w_{\mathrm{ij}}}^{2}-x{\left(\sum_{j=1}^{n}w_{\mathrm{ij}}\right)}^{2}}{n-1}}}$$

In the formula, “*n*” represents the number of spatial units (such as province or city). “*x*_*j*_” represents the Hemiptera insect number in the region numbered *j*. “*w*_*ij*_” is a weight matrix element to measure the spatial relationship of Hemiptera insects. If “*Z* (Gi)” is positive and significant, it indicates that the number of the Hemiptera insects around the study area is large and the area is a hot spot area, otherwise, it is a cold spot area. Hot spot analysis can better reflect the high‐value and low‐value agglomeration effects of the Hemiptera insects in a certain area.

#### 2.2.2 Standard deviation ellipse

The standard deviation ellipse is a classical algorithm for spatial statistical analysis proposed by American scholar Lefever (1926), which can better measure the direction and distribution characteristics of the data. It can effectively reveal the overall geographical elements' spatial distribution through the center point, semi‐major axis, and semi‐minor axis (Xiong et al., 2018). In this study, the center point reflects the relative position of the Hemiptera insect spatial distribution in the study area, indicating the centroid of the Hemiptera insect spatial distribution. The semi‐major axis reflects the Hemiptera insect data distribution direction in the study area. The semi‐minor axis represents the Hemiptera insect data distribution range. When the values of semi‐major axis and semi‐minor axis are oblateness, it can better reflect the spatial distribution pattern of Hemiptera insects in the study area; the larger the value, the more concentrated the spatial distribution (Li, Li, et al., 2021; Li, Liu, et al., 2021; Li, Zheng, & Wang, 2021). The standard deviation ellipse algorithm can reveal the spatial distribution characteristics of Hemiptera insects from multiple angles. We used the first level of standard deviation as a parameter; 63% of the data can be included.

#### 2.2.3 Geographical detector

We used the geo‐detector to analyze the characteristic factors affecting the spatial distribution of Hemiptera insects in China based on six major agricultural natural divisions. Species have latitude gradient diversity (Perez et al., 2016). China has a vast territory, spanning nearly 50 latitudes from north to south. Bingwei Huang (1959) divided China into 38 agricultural natural regions based on the classification of temperature zones and humidity levels. Most crops can only grow actively when the daily average temperature remains stable at or above 10°C. Therefore, the number of consecutive days with a daily average temperature above 10°C is typically referred to as the growing season. The sum of daily average temperatures within the growing season is known as accumulated temperature. The accumulated temperature of a region reflects its heat conditions. Based on the distribution of accumulated temperature, China is divided into five temperature zones and a special Qinghai‐Tibet Plateau region. Different temperature zones exhibit variations in heat, duration of the growing season, agricultural practices, and crop types. According to the accumulative temperature, China is divided into six major agricultural natural divisions: tropics, subtropics, warm temperate, mid‐temperate, cold cap, and Qinghai–Tibet Plateau (Huang, 1959). The cold cap has no distribution data for the Hemiptera insects.

Geographical detector is a new model of spatial statistics. Assuming that Hemiptera insects in different spatial positions have consistent changes with their influencing factors, this factor has a significant effect on the spatial distribution of Hemiptera insects. We used the model to detect the driving factors affecting the spatial distribution of Hemiptera insects. The model expression is as follows (Wang & Xu, 2017):

(6) $$\mathrm{PR}=1‐\frac{\sum_{i=1}^{L}N_{i}\sigma_{i}^{2}}{N_{i}\sigma^{2}}$$

In the formula, *i* = 1, 2, 3, …, L, it is the stratification of candidate factors. PR is the influence value of the factors on the spatial distribution of Hemiptera insects, and the value range is [0, 1]. The larger the value, the greater the influence of the factors on the spatial distribution of invasive organisms. $N_{i}$ is the candidate factor layer numbered *i*, and $\sigma_{i}^{2}$ and $\sigma^{2}$ are the variance of the candidate factors for layer i and the full layer, respectively.

## 3 RESULTS

### 3.1 Spatial agglomeration and spatial trend of the Hemiptera insects

For the global spatial autocorrelation, the Global Moran's *I* index of the Hemiptera insects on the provincial scale was 0.381, and the *Z*‐score was 2.07. The Global Moran's *I* index on the municipal scale was 0.12, and the *Z*‐score was 1.97, which was statistically significant (*p* < .05). It shows that the Hemiptera insects have positive spatial correlation in China, and there is spatial agglomeration, not random distribution. When the Hemiptera insects in a certain area are abundant, the Hemiptera insects in the surrounding area are also rich. The azimuth angle of the Hemiptera insect spatial distribution is 67.559°, the oblateness is 1.39, and the center position is 109.78804E, 30.211407 N. The ellipse with a semi‐major axis of 1453.99 km and a semi‐minor axis of 1043.91 km can cover about 63% of the Hemiptera insect spatial distribution points. The results show that the spatial agglomeration of Hemiptera insects is obvious, ranging from southwest to northeast, covering most provinces in China, such as Yunnan, Guangxi, Sichuan, Hunan, Hubei, Guangdong, Henan, Hebei, Zhejiang, Fujian, and Jiangxi.

**FIGURE 1:**
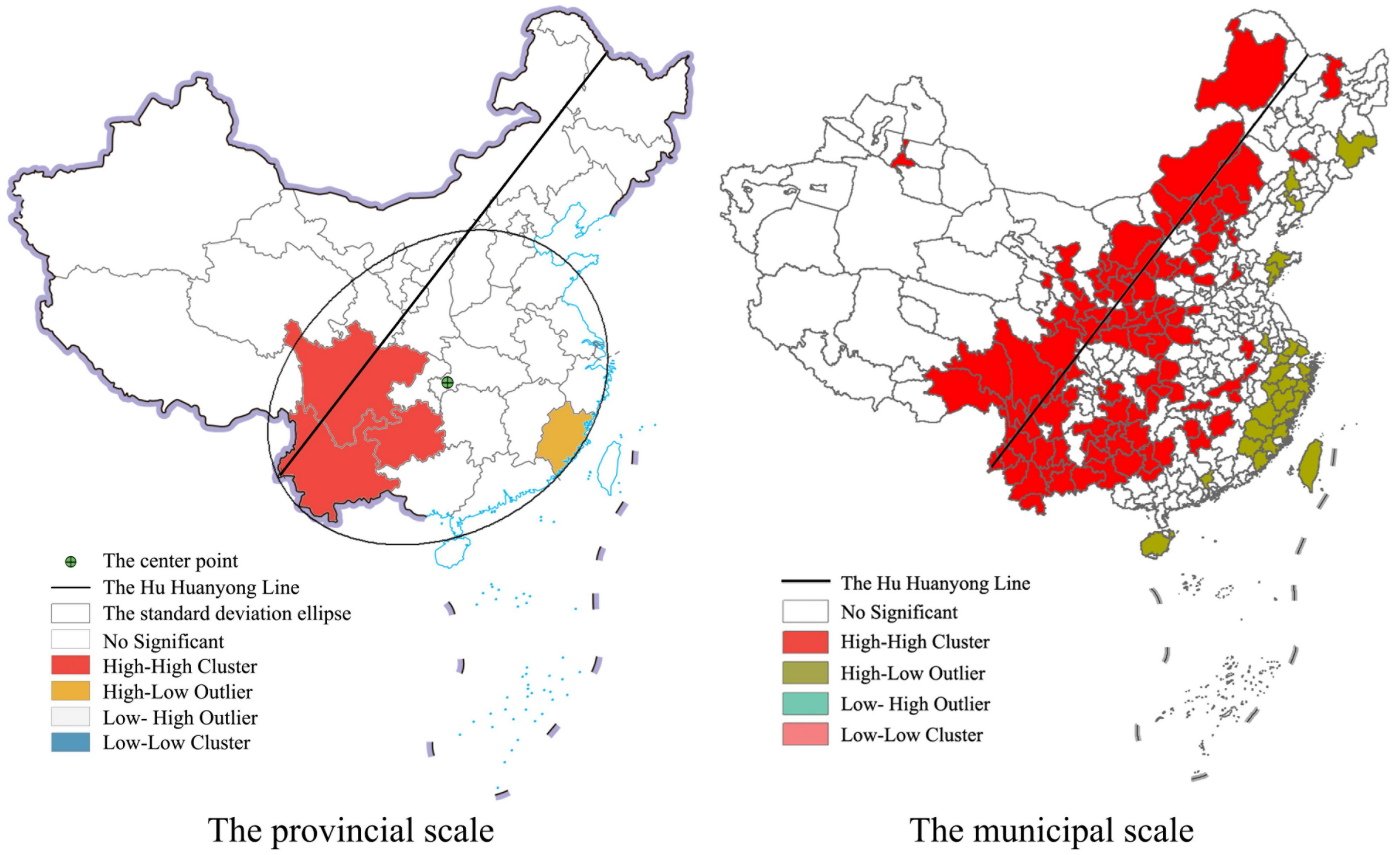
Local spatial distribution pattern of Hemiptera insects in China.

The results of the local spatial autocorrelation are shown in Figure 1. For the provincial scale, the Hemiptera insects have a clear spatial agglomeration. The HH type (high‐high cluster) is mainly distributed in the Yunnan–Guizhou–Sichuan group in Southwest China, showing a sheet distribution. The Hemiptera insect number in this area is generally large, showing a significant positive spatial correlation. The HL type (high‐low outlier) is mainly distributed in Fujian Province, and the Hemiptera insect number in Fujian Province is significantly higher than that in the surrounding provinces. For the municipal scale, the Hemiptera insects also have an obvious spatial agglomeration. The HH type is mainly distributed on both sides of the Hu Huanyong Line. The HL type is mainly distributed along Zhejiang‐Fujian, Taiwan, Hainan, Shandong, southern Jilin, and central Liaoning. In general, the Hemiptera insect spatial distribution in China shows a basic pattern of high in the southwest‐northeast, mainly distributed along the Hengduan Mountains, the Wuyi Mountains, the Greater Khingan Mountains, the Qinling Mountains, and the Tianshan Mountains, these areas are highly consistent with the spatial distribution of national key ecological function reserves and national parks.

### 3.2 Hot spot analysis of the Hemiptera insects

**FIGURE 2:**
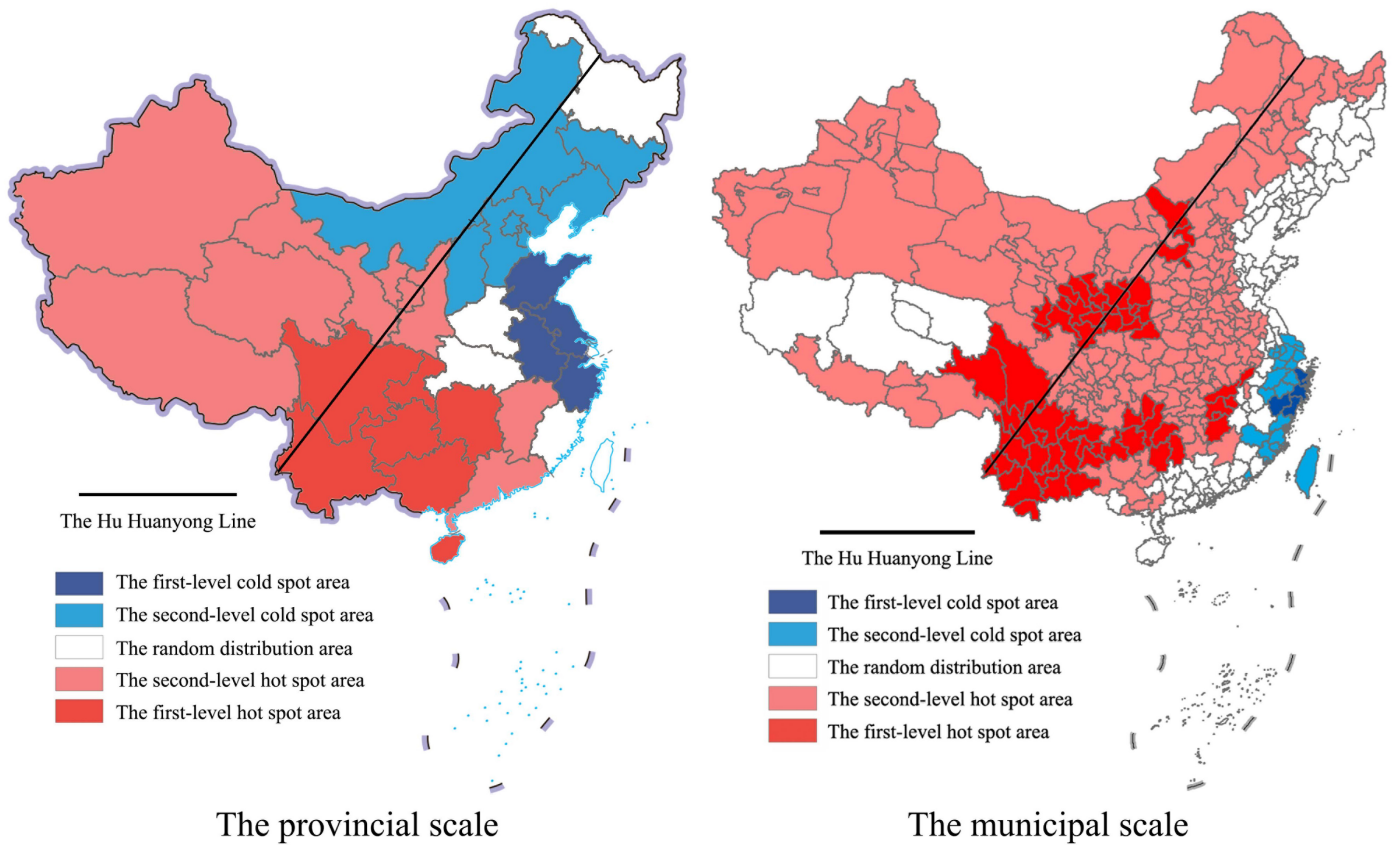
The spatial hot spot distribution of Hemiptera insects.

According to the value and significance level of the Getis‐Ord Gi index, the Hemiptera insects distribution area was divided into five categories by the natural breaks (Jenks) method in ArcGIS 10.1: The first‐level hot spot area (hot spot high concentration area), the second‐level hot spot area (hot spot low concentration area), the random distribution area, the second‐level cold spot area (cold spot low concentration area), and the first‐level cold spot area (cold spot high concentration area). The results are shown in Figure 2. For the provincial scale, the first‐level hot spot areas include Yunnan, Guangxi, Sichuan, Chongqing, Guizhou, Hunan, and Hainan. The first‐level cold spot areas include Jiangsu, Anhui, Zhejiang, and Shandong. For the municipal scale, the first‐level hot spot areas are mainly distributed in the southwest region, along the Qinling Mountains, the west side of the Wuyi Mountains, Yinshan, and other cities along the route. The first‐level cold spot areas are mainly distributed on the southeast coast.

### 3.3 Ecological geographical division of the Hemiptera insects

**FIGURE 3:**
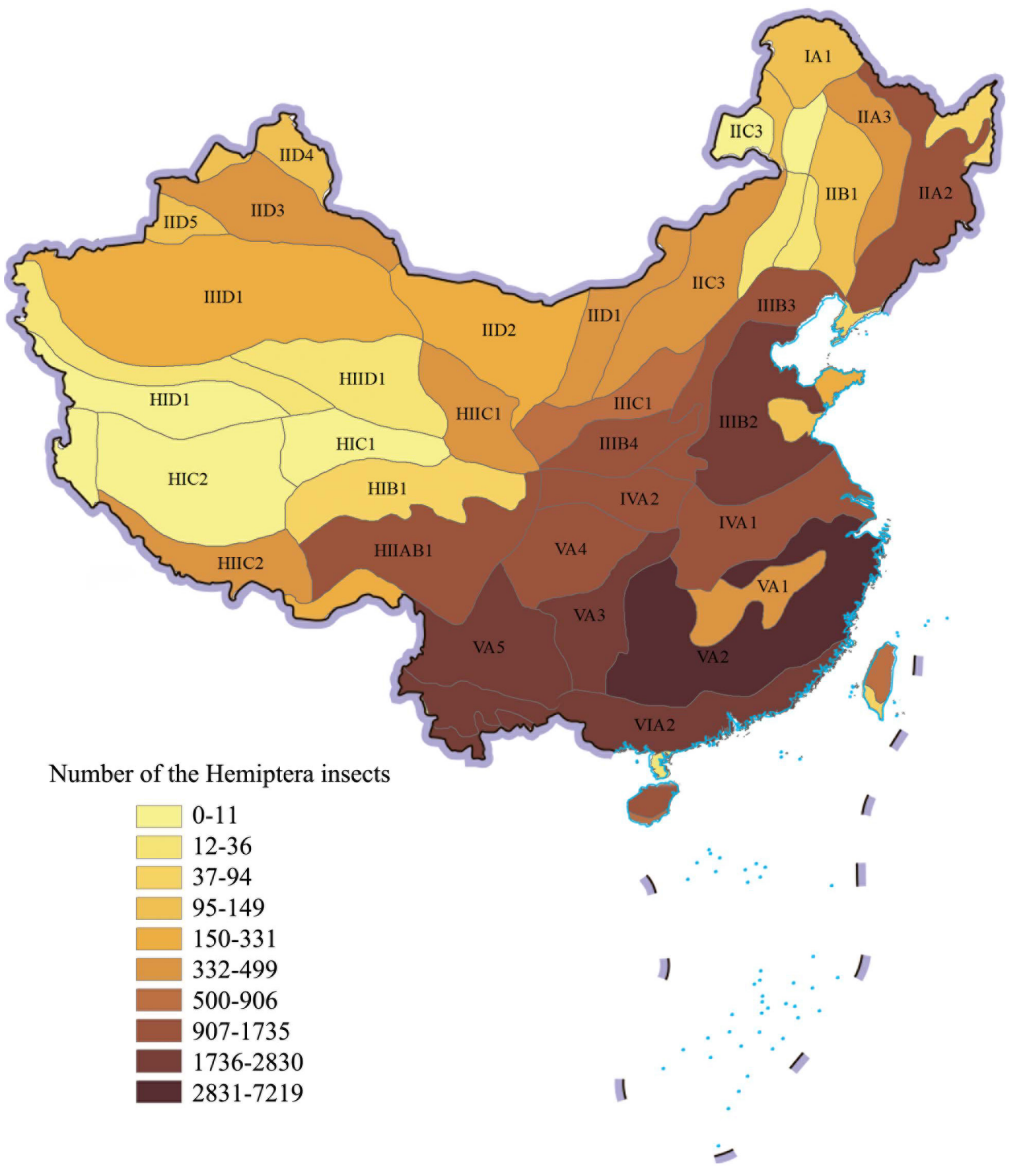
The spatial distribution of Hemiptera insects based on the eco‐geographical division.

We made the Hemiptera insects' eco‐geographical division in China, as shown in Figure 3. The description of the region code in Figure 3 is shown in the Table 1. The spatial distribution of Hemiptera insects has significant spatial differences in 48 ecological geographical divisions, showing a basic pattern of high in the south and low in the north, and high in the east and low in the west. Its center is located in the Guizhou Plateau. The top 10 eco‐geographical divisions with high proportion of the Hemiptera insects are as follows: the Jiangnan and Nanling Mountains (18.59%), the Fujian–Guangdong–Guangxi Hilly Plain (7.29%), the Yunnan Plateau (6.94%), the Guizhou Plateau (6.55%), the Valley Hills in Southern Yunnan (6.01%), the North China Plain (5.41%), the Hanzhong Basin (4.47%), the Sichuan Basin (4.06%), the Qionglei Mountain and Hills (3.97%), and the Eastern Mountains of the Northeast China (3.76%). The areas with less distribution of the Hemiptera insects are the Southwestern Songliao Plain (0.06%), the Northern Kunlun Mountains (0.06%), the Qaidam Basin (0.05%), the Ali Mountains (0.02%), the Qiangtang Plateau Lake Basin (0.01%), and the Kunlun Alpine Plateau (0.01%).

**TABLE 1:** Description of the region code.

Region code	Description
IA1	The Greater Khingan Mountains
IIA2	Eastern mountains of northeast China
IIA3	Northeast eastern Piedmont Plain
IIB1	Central Songliao Plain
IIC3	Eastern Inner Mongolia High Plain
IID1	Western Inner Mongolia High Plain and Hetao
IID2	Alxa and Hexi Corridor
IID3	Junggar Basin
IID4	Altai Mountain and Tacheng Basin
IID5	Yili basin
IIIB2	North China Plain
IIIB3	Mountains and hills of North China
IIIB4	Jinnan Guanzhong Basin
IIIC1	Shanxi central Shaanxi northern Gandong plateau hilly
IIID1	Tarim and Turpan basins
IVA1	Huainan and the middle and lower reaches of the Yangtze River
IVA2	Hanzhong basin
VA1	Jiangnan hills
VA2	Jiangnan and Nanling Mountains
VA3	Guizhou plateau
VA4	Sichuan basin
VA5	Yunnan Plateau
VIA2	Fujian Guangdong–Guangxi hilly plain
HIB1	Golonaku hilly plateau
HIC1	Qingnan Plateau‐wide valley
HIC2	Qiangtang Plateau Lake basin
HID1	Kunlun high mountain plateau
HIIA/B1	Sichuan Xizang east high mountains and deep valleys
HIIC1	Qingdong Qilian Mountain
HIIC2	Southern Tibet mountains
HIID1	Qaidam Basin

For the temperature belt, the Hemiptera insect distribution proportion from high to low is as follows: mid‐subtropical region (38%), warm temperate region (16.26%), mid‐temperate region (10.78%), marginal tropical region (10.21%), southern subtropical region (9.02%), northern subtropical region (7.42%), plateau temperate region (5.68%), mid‐tropical region (2.04%), cool temperate region (0.36%), and sub‐cold plateau region (0.23%). For the arid and humid region, the Hemiptera insect distribution proportion from high to low is humid region (72.83%), semi‐humid region (13.24%), semi‐arid region (5.77%), arid region (4.59%), and humid/semi‐humid region (3.57%). The Hemiptera insect distribution proportion in humid and semi‐humid region totaled 89.64%. It shows that temperature and precipitation have an important influence on the spatial distribution of Hemiptera insects, and the spatial distribution has obvious differentiation characteristics. According to the distribution characteristics, zoning and grading management can be implemented in a targeted manner, which has practical significance for the high‐quality protection and resources sustainable utilization of the Hemiptera insect biodiversity, and is conducive to the efficient prevention and control of pests.

### 3.4 Geo‐detector results analysis

We used the natural fracture method for discretization and divided the 11 impact factors into 10 levels. Furthermore, we used the geo‐detector tool to detect the factors affecting the spatial distribution of Hemiptera insects, and the PR values of factors are shown in Table 2. The results showed that the spatial distribution of Hemiptera insects in China was affected by multiple natural and socio‐economic environmental factors. The PR values of different factors and the dominant factors of different agricultural natural divisions were significantly different.

**TABLE 2:** The PR value of the factors affecting the Hemiptera insects' spatial distribution characteristics in China.

	X_1_	X_2_	X_3_	X_4_	X_5_	X_6_	X_7_	X_8_	X_9_	X_10_	X_11_
National scale	0.22	0.18	0.32	0.38	0.04	0.19	0.29	0.08	0.07	0.13	0.09
Tropics	0.13	0.51	0.42	0.25	0.29	0.20	0.29	0.13	0.48	0.17	0.11
Subtropics	0.09	0.15	0.15	0.14	0.07	0.07	0.13	0.07	0.12	0.13	0.12
Warm temperate	0.03	0.17	0.10	0.09	0.24	0.23	0.21	0.09	0.13	0.13	0.12
Mid‐temperate	0.18	0.08	0.10	0.09	0.10	0.09	0.05	0.14	0.13	0.31	0.31
Qinghai–Tibet Plateau	0.14	0.19	0.03	0.23	0.04	0.17	0.30	0.16	0.01	0.10	0.08

*Note*: The description of factors is shown in “2.1 Data sources”.

For the national scale, temperature seasonality (X4), isothermality (X3), annual precipitation (X7), and annual mean temperature (X1) have a great influence on the spatial distribution of Hemiptera insects—they are dominant factors. The dominant factors in the tropical region are mean diurnal range (X2), elevation (X9), isothermality (X3), max temperature of the warmest month (X5), and China's population spatial distribution kilometer grid dataset (X10). The dominant factors in the subtropical region are annual precipitation (X7), mean diurnal range (X2), isothermality (X3), temperature seasonality (X4), and China's population spatial distribution kilometer grid dataset (X10). Compared with the national scale and the tropical region, the natural and socio‐economic factors influence in the subtropical region is relatively balanced, which is related to the fact that the region is the main population and economic agglomeration area in China. The dominant factors in the warm temperate region are max temperature of the warmest month (X5), annual precipitation (X7), and precipitation seasonality (X6). For the mid‐temperate region, socio‐economic factors have a greater impact on the spatial distribution of Hemiptera insects than natural environmental factors—the dominant factors are China's population spatial distribution kilometer grid dataset (X10), China's GDP (gross domestic product) spatial distribution kilometer grid dataset (X11), and land use (X8). Obviously, temperature has a vital influence on the spatial distribution of Hemiptera insects.

To deeply explore the correlation between the driving factors of the spatial distribution of Hemiptera insects in China, we used the interactive detection of the geo‐detector to analyze the effects of the different factors' interaction on the spatial distribution of Hemiptera insects based on the national scale. The results are shown in Table 3.

**TABLE 3:** Interactive detection of factors affecting the spatial distribution of Hemiptera insects in China.

	X_1_	X_2_	X_3_	X_4_	X_5_	X_6_	X_7_	X_8_	X_9_	X_10_	X_11_
X_1_	0.22										
X_2_	0.58	0.18									
X_3_	0.55	0.56	0.32								
X_4_	0.58	0.68	0.63	0.38							
X_5_	0.38	0.50	0.58	0.62	0.04						
X_6_	0.47	0.55	0.62	0.57	0.45	0.19					
X_7_	0.41	0.62	0.67	0.61	0.51	0.59	0.29				
X_8_	0.38	0.40	0.47	0.50	0.25	0.36	0.42	0.08			
X_9_	0.38	0.42	0.56	0.60	0.22	0.39	0.47	0.25	0.07		
X_10_	0.43	0.48	0.54	0.55	0.33	0.40	0.49	0.35	0.26	0.13	
X_11_	0.36	0.47	0.50	0.57	0.27	0.37	0.49	0.27	0.25	0.26	0.09

*Note*: The description of factors is shown in “2.1 Data sources.” Light gray means two‐factor enhancement, and the others are nonlinear enhancement.

The interaction between the two factors has an enhancement effect on the Hemiptera insects' spatial distribution. X1&X4, X1&X7, X3&X4, and X4&X7 showed two‐factor enhancement (the two factors’ interaction is greater than single factor) and the other interactions showed nonlinear enhancement (the two factors’ interaction is greater than the two factors' sum effect). It shows that the different factors' interaction will greatly enhance the interpretation of the Hemiptera insects' spatial distribution. For example, the PR value reached 0.68 after the interaction of X2 and X4, and the PR value reached 0.6 after the interaction of X4 and X9. The interactive detection results of agricultural natural zoning are consistent with the national scale; the driving force of the two‐factor effect is much higher than that of the single factor. Therefore, the Hemiptera insects' spatial distribution in China is affected by multiple factors.

## 4 DISCUSSION

### 4.1 Mountains are hot spots region for the Hemiptera insects

In this study, we used spatial statistical analysis methods and geo‐detector tools to investigate the spatial distribution characteristics of Hemiptera insects in China. In general, the spatial differentiation of Hemiptera insects is remarkable, showing a basic pattern of high in the southwest‐northeast, high in the east, and low in the west. This is basically consistent with the importance pattern of biodiversity conservation in China (Wu & Meng, 2022), and there is a high degree of consistency with the spatial distribution of national key ecological function reserves and national parks.

Mountains are closely related to biodiversity (Liu et al., 2009; Tang et al., 2006), and are the hot spots and cradles of biodiversity (Quintero & Jetz, 2018). The role of mountains in affecting biodiversity is multifaceted, far‐reaching, and often indirect (Perrigo et al., 2020). Due to the combined effects of elevation and hydrothermal conditions, the species abundance in mountainous areas is significantly higher than that in plain areas (Zhang et al., 2022). Comparing the major mountain chains (Wang et al., 2004), the spatial distribution hot spots of Hemiptera insects in China are mainly in the southwestern region, coastal areas along the Qinling Mountains, the western side of the Wuyi Mountains, the Yinshan Mountains, the Liupan Shan, the Xuefeng Mountains, and the Nanling Mountains. For the ecological geographical zonation, the distribution center of the Hemiptera insects in China is located in the Guizhou Plateau. The Hemiptera insect number in the Jiangnan and Nanling Mountains, the Fujian–Guangdong–Guangxi Hilly Plain, the Yunnan Plateau, the Guizhou Plateau, the Valley Hills in Southern Yunnan, and the Eastern Mountains of Northeast China exceeded 51% of the total number of Hemiptera insects. This indicates that mountains are the hot distribution spots of Hemiptera insects in China, and mountainous areas are crucial for the Hemiptera insect diversity conservation. Other related studies also support this point (Li, Li, et al., 2021; Li, Liu, et al., 2021; Li, Zheng, & Wang, 2021).

### 4.2 Environmental factors' effects on the spatial distribution pattern of Hemiptera insects

Insects are poikilothermic animals, and their growth and development are closely related to the environment. Temperature has a crucial impact on insect physiology, reproductive capacity, and symbiotic bacteria (Chen & Ma, 2010; Ma & Ma, 2016; Xin et al., 2019), and the impact of climate change is becoming increasingly important (Chen & Ma, 2010; Newbold et al., 2015). In this study, the detection results of the geo‐detector model indicate that the spatial distribution of Hemiptera insects in China is impacted by the interaction of temperature, precipitation, land use, and socio‐economic factors. In relative terms, the natural environment is an important basis for the spatial distribution pattern of Hemiptera insects. The temperature seasonality, the isothermality, the annual mean temperature, and the annual precipitation are the dominant factors, which has an essential influence on the spatial distribution pattern of Hemiptera insects in China. Affected by hosts, land use has a key impact on insect biodiversity changes (Newbold et al., 2015). The results of geo‐detector show that land use has a great influence on the spatial distribution of Hemiptera insects in the mid‐temperate, tropical, and Tibetan Plateau regions. Although it was not the dominant factor, the direct interaction between land use and other influencing factors is the most intense. Related studies also showed this point (Quintero & Jetz, 2018).

Insects have the ability to spread and migrate but are affected by the natural environment, hosts, and human activities (Chen & Ma, 2010). The results of the interaction detection show that the factors' influence on the spatial distribution of Hemiptera insects is not independent—it is a synergistic enhancement of nonlinear enhancement and two‐factor enhancement. In particular, the direct interaction between natural environmental factors and socio‐economic factors is stronger than the interaction within the same type. The interaction between natural and socio‐economic factors has a significant positive strengthening effect on the spatial distribution of Hemiptera insects, indicating the complexity and diversity of the influencing factors on the spatial distribution of Hemiptera insects. The interaction between climate and mountains can form rich species diversity (Perrigo et al., 2020). In this study, the interactive detection results show that the interaction between climate and elevation factors has a significantly enhanced effect.

Species have latitudinal gradient diversity (Perez et al., 2016), and the spatial scale effect may have a key impact on the spatial stratification heterogeneity analysis (Song et al., 2020). The specific performance is that the factors that influence the spatial distribution characteristics of Hemiptera insects are different in space. The partition detection results show that the influence of natural environment is obviously different among agricultural divisions such as tropical, subtropical, warm temperate, mid‐temperate, and cold cap, while the socio‐economic factors are relatively stable, and different factors form a unique interaction mode. In the tropics, elevation, mean diurnal range, and isothermality have high PR values, which are the dominant driving factors. In the warm temperate, the mid‐temperate, and the Qinghai–Tibet Plateau, the PR values of factors are low. This may be due to the relatively stable temperature in the tropics. Tropical species are considered to be more sensitive to climate change than temperature species and have a narrower thermal niche (Newbold et al., 2020; Perez et al., 2016).

### 4.3 The Hu Huanyong line and the spatial distribution of Hemiptera insects

The Hu Huanyong Line is an important demarcation line for the Hemiptera insects in China. For the municipal scale, the local spatial autocorrelation analyses show that there is an obvious spatial agglomeration among the Hemiptera insects. The HH type (high‐high cluster) is mainly distributed on both sides of the Hu Huanyong Line, representing the spatial distribution characteristics of high in southwest‐northeast. It could be related to the fact that the Hu Huanyong Line is an important ecological transition zone in China. The Hu Huanyong Line is an important dividing line between natural patterns (such as climate and vegetation) and cultural patterns (such as population and transportation) (Ding et al., 2021). It is close to the second and third terrace demarcation lines of China's topography, the rainfall line of 400 mm in the north, and the rainfall line of 800 mm in the south. And the climate, hydrology, and vegetation have undergone a rapid transition (Wang et al., 1995).

The differences in insect biomass and richness are highly dependent on the environment (Uhler et al., 2021). Climatic conditions (such as temperature, precipitation, and cumulus) are important influences affecting ecological types and have an important impact on the spatial pattern of species richness (Quintero & Jetz, 2018). The natural environment such as temperature, precipitation, humidity, and vegetation along the Hu Huanyong Line shows obvious transition (Wang et al., 1995). In addition, agricultural intensification is considered to be the main driving force for insect reduction. Related scholars' studies have shown that agricultural intensification is rapidly reducing insect biodiversity (Klink et al., 2020; Ravena & Wagner, 2020). The Hu Huanyong Line is the demarcation line of farming–pastoral ecotone and agricultural production potential (Chen, 2018; Wang et al., 2019), and there are obvious differences in agricultural production methods on both sides. On the east side of the Hu Huanyong Line, the climate environment is suitable, the agricultural intensification degree is high, and human activities are abundant. On the west side, the vegetation is sparse, and human activities are weak.

However, our study still has some limitations. Firstly, using geo‐detector to explore the key factors affecting the spatial distribution characteristics of Hemiptera insects in China, the results can only analyze the factors' influence degree and cannot infer causality (Uhler et al., 2021). Secondly, in the process of spatial data discretization, it is necessary to combine professional experience and knowledge to improve the accuracy of the results. Thirdly, the species of Hemiptera insects are prosperous—there are great differences in host range, feeding habits, economic value, and requirements of temperature and humidity. Due to the limitation of the Hemiptera data sources, this research lacks research on the differences in widespread species, rare species, dominant species, and their temporal and spatial variation characteristics of the Hemiptera insects. In further research, the distribution of suborders or families should be considered in combination with agricultural natural divisions to improve the application value.

## 5 CONCLUSIONS

In this study, we used a variety of spatial analysis methods to explore the spatial distribution characteristics of Hemiptera insects and clarify the dominant factors affecting the spatial distribution of Hemiptera insects. The results show that the spatial differentiation characteristics of the Hemiptera insects in China are significant. The Hemiptera insects have obvious spatial agglomeration, and the Hu Huanyong Line is an important dividing line for the spatial distribution of Hemiptera insects in China. In general, the spatial distribution of Hemiptera insects in China shows a basic pattern of high in the south and low in the north, high in the east and low in the west, and mountains are hot spots region for the spatial distribution of Hemiptera insects. In addition, the spatial distribution of Hemiptera insects in China is affected by multiple factors such as natural and socio‐economic factors. The driving force of the two‐factor effect is much higher than that of the single factor. Natural and socio‐economic have formed a unique interaction pattern, which has a significant positive reinforcement effect on the spatial distribution of Hemiptera insects. The results of this study can provide scientific guidance for the implementation of hierarchical management in different regions to achieve high‐quality ecological stability, biodiversity conservation, and efficient pest control.

## AUTHOR CONTRIBUTIONS

**Zhipeng Li:** Conceptualization (equal); data curation (equal); formal analysis (lead); funding acquisition (lead); project administration (lead); visualization (equal); writing – original draft (lead); writing – review and editing (equal). **Xinyi Zhang:** Visualization (equal); writing – original draft (lead); writing – review and editing (equal). **Jian Zhao:** Conceptualization (equal); data curation (equal); funding acquisition (lead); project administration (lead); supervision (lead); writing – review and editing (equal). **Hong Chen:** Data curation (equal); writing – review and editing (equal). **Maofen Tian:** Visualization (equal); writing – review and editing (equal).

## CONFLICT OF INTEREST STATEMENT

The authors declare that they have no conflict of interest or personal relationships that could have appeared to influence the work reported in this paper.

## Data Availability

The data that support the findings of this study are openly available in Dryad at: https://doi.org/10.5061/Dryad.jq2bvq8h2. The temporary download URL: https://datadryad.org/stash/share/LUBUhV31ecBPTVEkZf10MSlfixtMotIrJFHPQoE1JuQ.
